# Advanced Techniques for Bone Restoration and Immediate Loading after Implant Failure: A Case Report

**DOI:** 10.3390/healthcare11111608

**Published:** 2023-05-31

**Authors:** Neculai Onică, Cezara Andreea Onică, Elena-Raluca Baciu, Roxana-Ionela Vasluianu, Mihai Ciofu, Mihail Balan, Gabriela Luminița Gelețu

**Affiliations:** 1 Specialist Oral and Maxillofacial Surgery, Private Practice, 700612 Iasi, Romania; nicuonica@yahoo.com; 2 Specialist Oral Surgery, Private Practice, 700612 Iasi, Romania; dr.cezaraonica@gmail.com; 3 Department of Implantology, Removable Dentures, Dental Technology, Faculty of Dental Medicine, University of Medicine and Pharmacy, “Grigore T. Popa”, 700115 Iasi, Romania; 4 Department of Surgery, Faculty of Dental Medicine, University of Medicine and Pharmacy “Grigore T. Popa”, 700115 Iasi, Romania; mihai.ciofu@umfiasi.ro (M.C.); mihail.balan@umfiasi.ro (M.B.); gabriela.geletu@umfiasi.ro (G.L.G.)

**Keywords:** dental implants, bone graft, immediate dental implant loading, interim dental prosthesis

## Abstract

The objective of this study was to report a clinical case of dental implant failure with significant bone loss that was treated using reconstructive surgical techniques. We present a 58-year-old man with a history of implant surgery and implant failure on the mandible. Data collected using cone beam computed tomography (CBCT) and intraoral scans were exported into Exoplan (exocad GmbH, Darmstadt, Germany), from which a standard tessellation file was obtained. To create a customized mandible mesh design, DentalCAD 3.0 Galway software (exocad GmbH, Darmstadt, Germany) was used. Based on guided bone regeneration, the method involved bone reconstruction and the application of a custom titanium mesh. The bone mix was obtained by combining a xenograft (Cerabone, Bottis biomaterials Gmbh, Zossen, Germany), an allograft (Max Graft, granules Bottis biomaterials Gmbh, Zossen, Germany), and an autograft. The titanium meshes were fixed to the bone using self-drilling screws and covered with a resorbable membrane. Immediately after surgery, an impression was recorded, and the next day, the patient received a milled polymethyl methacrylate interim denture. Based on our case study, the presented custom-made implant can be considered a temporary solution, during which guided bone regeneration is expected to take place.

## 1. Introduction

Implant location and primary stability, soft tissue shape recovery, and other crucial aspects of a successful implantation restoration in oral implantology are all influenced by the quantity and quality of alveolar bone at the implant site [1]. After tooth loss, the alveolar bone often experiences secondary resorption and atrophy, and over time, the breadth and height of the alveolar ridge diminish, making them unsuitable for implant placement. Furthermore, when placed implants are lost due to periimplantitis, the bone loss becomes dramatic. 

A crucial aspect of oral implantology is the formation of alveolar bone. Alveolar bone defect repair can be clinically accomplished using a variety of techniques, including guided bone regeneration (GBR), onlay bone grafting, bone extrusion, bone splitting, and distraction osteogenesis. Bone repair is necessary to address anatomical and prosthetic needs that cannot be met by short or slanted implants [2]. Consequently, a flexible regeneration approach is essential when treating locations with uneven morphologies, impaired thickness and height, and soft tissues that are thin, weakly keratinized, and prone to dehiscence. 

In order to meet the initial stability and osseointegration criteria of implantation, Von Arx et al. have been stabilizing autologous bone grafts for regional alveolar ridge repair with titanium (Ti) mesh since 1996 [3]. 

Numerous bone deformities can be managed using 3D-printed custom titanium meshes due to their exceptional precision; this makes them ideal for guided bone regeneration (GBR), since they promote strong, long-lasting osteogenesis, achieve an adequate amount of space, and permit simultaneous bone augmentation in the horizontal and vertical axes [4]. 

Corrosion resistance and cytotoxicity are two attributes of material biocompatibility. Due to its low electrical conductivity, titanium is susceptible to electrochemical oxidation, which can produce a passive and inert oxide layer [5]. This layer may be preserved under the pH of the human body, giving titanium high and long-lasting corrosion resistance [6]. Titanium products release a small number of ions to the surrounding tissues, and have no noticeable impact on the relative growth rate of human cells [7,8]. A pseudo-periosteum, or a thin layer of soft tissue, was frequently found to cover the surface of newly produced bone after a titanium mesh was used to repair an alveolar ridge, as reported by Cucchi et al. [9].

Customized Ti mesh has some drawbacks; specifically, it has a non-antibacterial effect, a second surgery is required to remove it, and Ti radiopacity may affect the imaging results of future X-ray examinations [10,11,12]. 

Compared to a traditional approach, customized Ti mesh, with its smooth, round, and blunt shape, can result in less membrane exposure. However, in certain instances, Sagheb et al. [13] found that the exposure rate of a customized mesh may reach an average of 20% or higher due to its inherent stiffness.

By using an appropriate surgical approach along with autologous bioactive materials, a faster healing time and reduced exposure of the titanium mesh can be attained [14,15,16,17,18]. In such situations, titanium meshes have shown remarkable biocompatibility and mechanical properties, indicating their efficacy as a barrier membrane for GBR to heal bone defects [19,20].

The aim of this study was to provide a clinical example of dental implant failure with important bone loss that was treated using reconstructive surgical techniques.

## 2. Case Report

**Figure 1:**
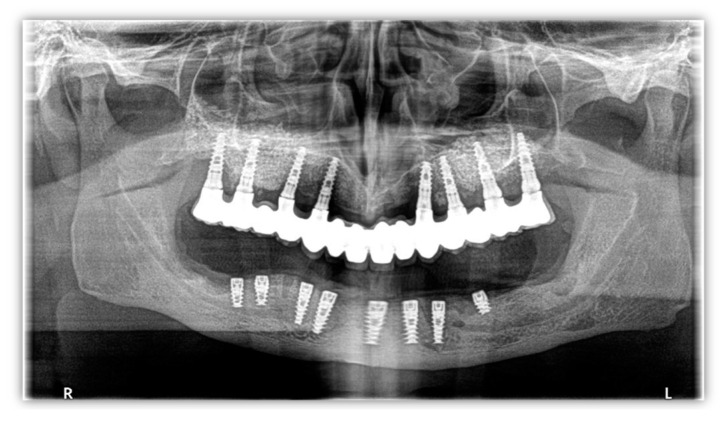
Orthopantomography image from 2014.

A 58-year-old man with a history of implant surgery (Figure 1) and implant failure on the mandible, leading to significant bone loss, was referred to us for bone restoration and functional loading.

A clinical examination confirmed the loss of three implants in the right mandible side and one implant in the left side.

**Figure 2:**
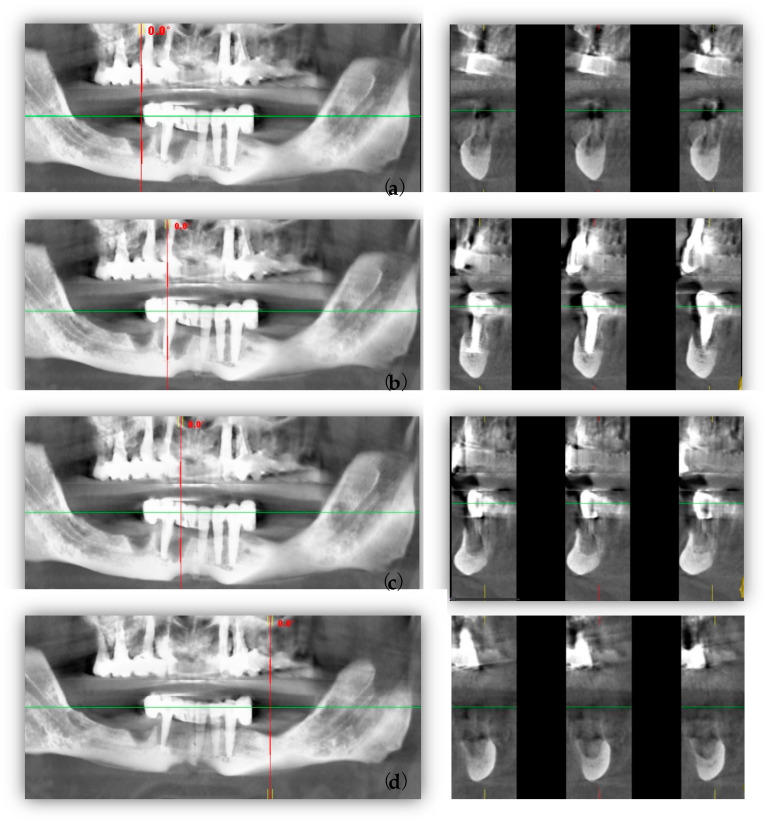
Pre-surgical CBCT images: (**a**) panoramic (green line) and cross-sectional (red line) views of bone defect after implant failure; (**b**) panoramic (green line) and cross-sectional (red line) views of 4.3 implant; (**c**) panoramic (green line) and cross-sectional (red line) views of bone defect after implant failure; (**d**) panoramic (green line) and cross-sectional (red line) views of bone defect after implant failure.

Cone beam computed tomography (CBCT) revealed that the three remaining implants in the anterior left mandible side had appropriate implantation and decent appeal (Figure 2a–d).

**Figure 3:**
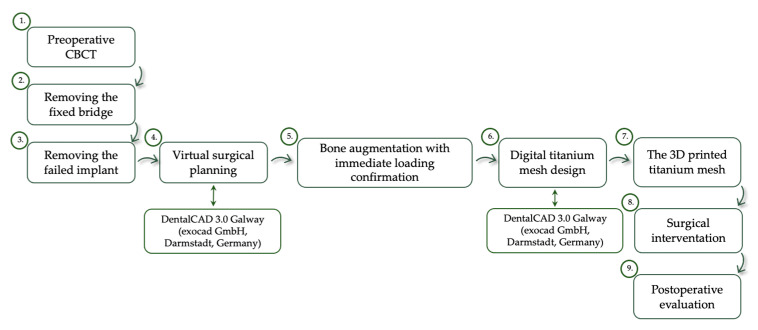
The surgical treatment workflow.

In order to protect these implants, we decided to remove the fixed bridge and to extract the compromised implant from the patient’s right mandible side. The treatment plan steps are presented in Figure 3.

After informing the patient of the diagnosis, prognosis with and without therapy, specific therapeutic steps, and the advantages, procedure-specific risks, and probable adverse effects of treatment, informed consent was acquired.

Due to personal concerns and biological considerations, the patient’s surgery was scheduled for two months following implant removal.

The data collected via CBCT and intraoral scans (Medit i700, MEDIT corp. 8, Seoul, Republic of Korea) were exported into Exoplan (exocad GmbH, Darmstadt, Germany), from which a standard tessellation (.STL) file was obtained. 

**Figure 4:**
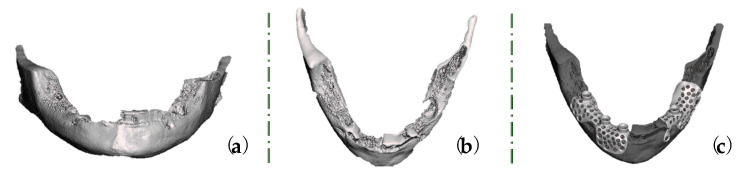
Virtual images of bone loss in (**a**) panoramic view and (**b**) axial view. (**c**) The designed custom-made titanium meshes.

The STL file was exported into DentalCAD 3.0 Galway software (exocad GmbH, Darmstadt, Germany) to reconstruct the bone loss and to design two customized meshes for each mandible side (Figure 4a–c). 

The custom-made titanium meshes were designed with a 0.6 mm thickness, holes with a 2 mm diameter on the entire smoothened surface, rounded edges, and prosthetic connections (three for the right mandible side and one for the left side) and were compatible with Nobel Biocare multi-unit abutments (Nobel Biocare Services AG, Zürich-Flughafen, Switzerland). 

The obtained STL files were sent to a 3D printing machine (3D Laser Metal Fusion Technology, mysint100 SISMA, S.p.A., 36013, Piovene Rocchette Italy) to be fabricated using titanium alloy powder (PowderRange Ti64, Carpenter Technology Corporation, Reading, PA, USA).

Acid etching, plasma cleaning, and sterilization were performed before packing and shipping the mesh.

The patient’s blood tests were in the normal range. At induction, the patient received an intravenous non-steroidal analgesic (Ketorol, Dr. Reddy’s laboratories, București, Romania) and a loading dose of 2 g of the antibiotic amoxicillin.

The patient rinsed with a 0.2% solution of chlorhexidine (Curasept, Curaden Healthcare) for 1 min, and a sterile surgical drape was applied to disinfect the surgical site. Local anaesthesia was induced using an articaine solution (4%) with epinephrine (1:100,000; Ubistein, 3 M ESPE). The full-thickness buccal and lingual flaps were raised to reveal the whole bone defect after making a mid-crestal incision (surgical blade No. 15c) with vertical releasing incisions. The emergence of the mental nerve was then bilaterally determined.

By releasing the incisions and dividing the periosteum, the flaps were coronally stretched to provide full closure with a passive suture above the titanium implant. Bone flakes were bilaterally harvested from the extern oblique line using a safe scraper.

**Figure 5:**
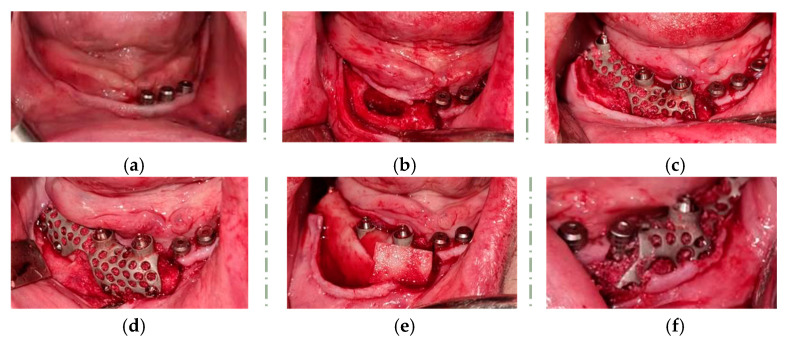
Intraoperative images: (**a**) the residual alveolar ridge aspect; (**b**) flap elevation; (**c**) right side—custom titanium mesh application; (**d**) right side—fixing the custom titanium mesh with the screws; (**e**) right side—covering the custom titanium mesh with resorbable membrane; (**f**) left side—custom titanium mesh application.

The bone mix was obtained by combining a xenograft (Cerabone 0.5–1 mm, Bottis biomaterials Gmbh, Zossen, Germany), an allograft (Max Graft, granules Bottis biomaterials Gmbh, Zossen, Germany), and an autograft (bone flakes harvested from the external oblique ridge). The titanium meshes were fixed to the bone using self-drilling screws (2/5 mm and 2/7 mm, Medicon eG, Tuttlingen, Germany) and then covered with a Mucoderm resorbable membrane (Bottis biomaterials Gmbh, Zossen, Germany) (Figure 5a–e).

**Figure 6:**
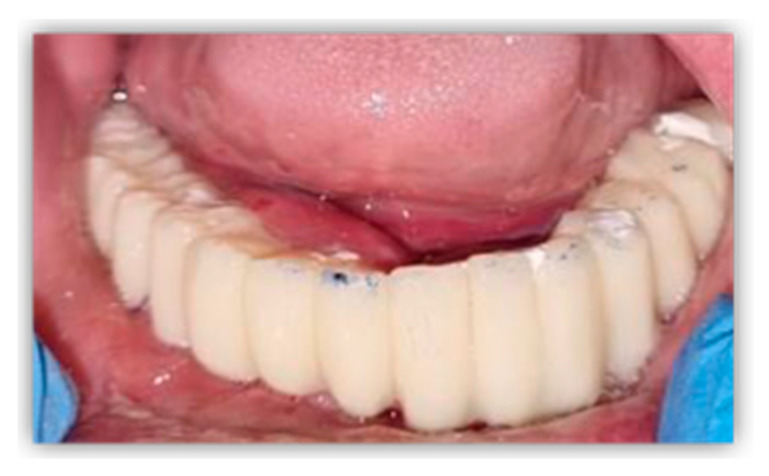
Intraoral aspect of milled PMMA interim denture.

Immediately after surgery, a direct impression was recorded using open-tray and polyether impression material (Impregum, 3 M ESPE, St. Paul, MN, USA). Bite registration was performed using an occlusion rim, and the next morning, the patient received a milled polymethyl methacrylate (PMMA) interim denture, restoring their dental functions and facial aesthetic. Using screws, the provisional prosthetic restoration was fixed to the level of the remaining dental implants and mesh prosthetic connections (Figure 6). 

The day following surgery, the patient took 2 g (oral dosage, 1 g twice a day) of amoxicillin and clavulanic acid (Augmentin, Glaxo Wellcome Production ZI, Peyenniere, 53100 Mayenne, France) each day for the next 6 days. The patient was instructed not to brush the surgical site for three weeks or eat soft foods and to practice proper oral hygiene, which included twice-daily washing with 0.2% chlorhexidine and applying 0.2% chlorhexidine gel to the wounds.

**Figure 7:**
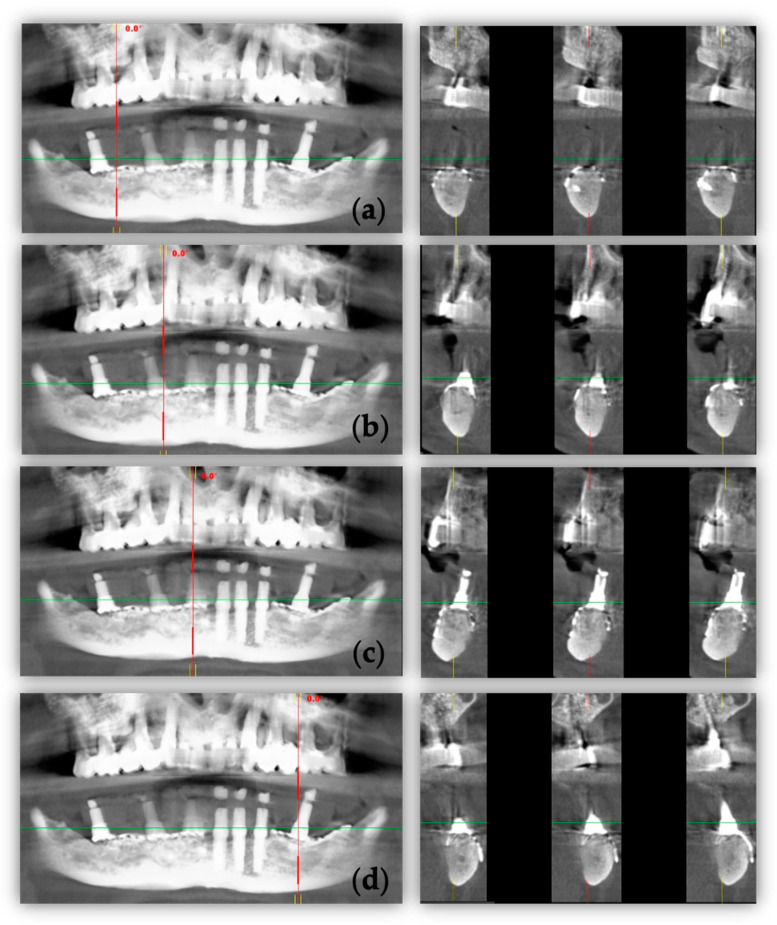
Six-month postsurgical CBCT images: (**a**–**d**) panoramic (green lines) and cross-sectional (red lines) views.

Following weekly examinations in the first month, the patient has since been followed-up once a month. No exposure or infection have been documented. A six-month CBCT scan dataset was acquired to confirm the occurrence of bone augmentation (Figure 7a–d). 

## 3. Discussion

Severe mandibular bone resorption caused by implant failure limited our therapy options. The reconstruction of lost bone usually takes time before implants can be placed and function restored. For the specific clinical condition of our patient, we selected a therapeutic method that would enable us to reconstruct the bone and offered the possibility of immediate loading. The digital component had a major influence in the stability of this case. 

Using cross-sectional imaging and digitalized research models, a properly restored alveolar ridge can be digitally created according to the patient’s arch shape and predicted implant position using computer-aided design (CAD) software following extensive preoperative evaluation [21].

In addition, if the custom titanium mesh has the appropriate characteristics, such as thickness, stiffness, and fitting, it can be combined with prosthetic components to attempt immediate loading.

According to Rakhmatia et al. [22], the thickness of the titanium mesh may influence the overall quantity of the formed bone, and the size of the pores may affect the produced bone and soft tissues.

Titanium mesh thickness varies from 0.1 to 0.6 mm and is correlated with its mechanical properties. In most clinical situations, 0.2 mm titanium meshes are used. At this thickness, proper flexibility and stiffness can be ensured to prevent tissue rupture while maintaining space and protecting the grafts.

Compared to standard titanium meshes, printed meshes have superior stiffness but lack plasticity [23]. To preserve bone regeneration space in the alveolar reconstruction area and to carry the load of dental arches, a thicker titanium mesh should be employed in GBR. To support immediate loading, we designed a mesh with a 0.6 mm thickness.

Pore size affects titanium mesh bone augmentation efficiency. The titanium mesh’s pores may help improve the grafts’ metabolic processes and ensure adequate blood supply [24].

Celletti et al. [25] found that a titanium mesh without holes was exposed three weeks after surgery. Nonetheless, titanium mesh holes and bone formation remain controversial. The holes make selective cell isolation difficult, and soft tissue often forms below the mesh. 

According to Gutta et al. [24], a titanium mesh with a large diameter (1.2 mm) encouraged greater bone regeneration and successfully inhibited soft tissue development compared to a titanium mesh with a small diameter (0.6 mm). The enhanced blood supply and oxygen and nutrient diffusion caused by the big aperture may be responsible for this phenomenon. On the other hand, Her et al. [26] revealed in their study that a titanium mesh with a large diameter (>2 mm) may promote greater soft tissue formation on the surface of the new bone than a titanium mesh with a small diameter.

GBR using a titanium mesh has a great capacity to anticipate osteogenesis, and both horizontal and vertical bone augmentation may be achieved during the procedure with either delayed or simultaneous implantation. Most research has shown that the delayed implantation technique of bone augmentation results in an average augmentation of 4–5 mm in bone width and 5–7 mm in bone height [13,27,28,29,30]. 

Compared to other methods, bone resorption due to infection is rare in the application of a titanium mesh [31,32,33]. Nevertheless, Zhang et al. [34] demonstrated that for a single anterior tooth defect, throughout a 41-month follow-up period following implant placement, the labial bone plate underwent an average of −0.81 ± 1.00 mm vertical absorption, with bone atop the implant absorbing at a mean of −0.13 ± 1.19 mm in the horizontal dimension. Over an 88-month follow-up period following implantation, Poli et al. [35] found that the average mesial and distal bone resorption values were at 1.743 mm (standard deviation: 0.567) and 1.913 mm (standard deviation: 0.71), respectively, for a wide range of GBR with titanium. Hence, when using a titanium mesh for bone augmentation, it is also necessary to take bone resorption into account.

In comparison to existing protocols, our procedure offers a distinct advantage, whereby bone addition and functional loading are carried out within a single intervention. However, it is important to note that our method incurs certain risks, including excessive flap elevation, wound dehiscence, exposure of the custom-made implants, and the potential for infection.

Our report may be limited by the fact that it focused exclusively on the treatment of a single patient with bone loss caused by periimplantitis. Given this constraint, it is imperative to apply our method to various pathologies, such as oncology and trauma, to establish an effective protocol.

## 4. Conclusions

Currently, we can simulate our complete treatment procedure with the help of specialized software, which allows us to restore missing hard tissue based on a prosthetic solution and future implant characteristics and placements.

According to our case report, the presented custom-made implant can be considered a temporary solution, during which guided bone regeneration is expected to take place.

## Data Availability

Not applicable.
